# Western Agarose Native GeELution (WANGEL) with beta-amyloid and tau: Novel method to elute proteins or peptides using native agarose gels followed by Lumipulse assay

**DOI:** 10.1016/j.mex.2022.101779

**Published:** 2022-07-06

**Authors:** Dhwani S. Korde, Christian Humpel

**Affiliations:** Laboratory of Psychiatry and Experimental Alzheimer's Research, Medical University of Innsbruck, Innsbruck 6020, Austria

**Keywords:** AD, Alzheimers disease, CSF, cerebrospinal fluid, Ab(42), beta-amyloid, 42 amino acids, Ab(40), beta-amyloid, 40 amino acids, WANGEL, Western Agarose Native GeELution, PVDF, polyvinylidene fluoride, CJD, Creutzfeld Jakob Disease, Western blot, Agarose gel, Elution, Tau, Beta-amyloid, Lumipulse ELISA, Alzheimer's disease

## Abstract

Alzheimer´s disease is characterized by hyperphosphorylated tau neurofibrillary tangles and beta-amyloid plaques. Both molecules can be easily measured in human fluids or tissue extracts by immunoassays. However, the different molecular weight species can only be differentiated on Western Blot gels. Analysis of native proteins from polyacrylamide gels is also not well characterized. Hence, we developed a modified method to elute proteins or peptides from native agarose gels. Initially, full-length tau (60 kDa) and beta-amyloid(42) (4 kDa) were separated on a Western Blot gel and eluted from native agarose gels (WANGEL) using an elution system inside a polypropylene tube. The eluates were analyzed with the Lumipulse immunoassay. Both molecules were successfully eluted into 1% agarose gels to the cathode and were detected in the eluate. Additionally, tau was eluted from mouse cortical extracts, but was below the detection limit when eluted from human cerebrospinal fluid. Beta-amyloid(40) was eluted from CSF extracts and detected by Lumipulse. In cortical extracts taken from transgenic mice (APP_SweDI) beta-amyloid(42) was detectable as a native peptide and small oligomeric aggregates. Taken together, our novel WANGEL method enables fast, easy and cheap elution of protein/peptides from polyacrylamide/agarose gels with a subsequent analysis by Lumipulse immunoassay.

Three bullet points:

• Beta-amyloid and tau are major hallmarks in Alzheimer´s disease and are established cerebrospinal fluid biomarkers.
• Lumipulse is a method to measure beta-amyloid and tau in cerebrospinal fluid in the pg/mL range.
• Western Blot and our novel combined native agarose method (WANGEL) allows an easy and fast determination of the molecular size in combination with Lumipulse.

## Graphical Abstract

**Figure:**
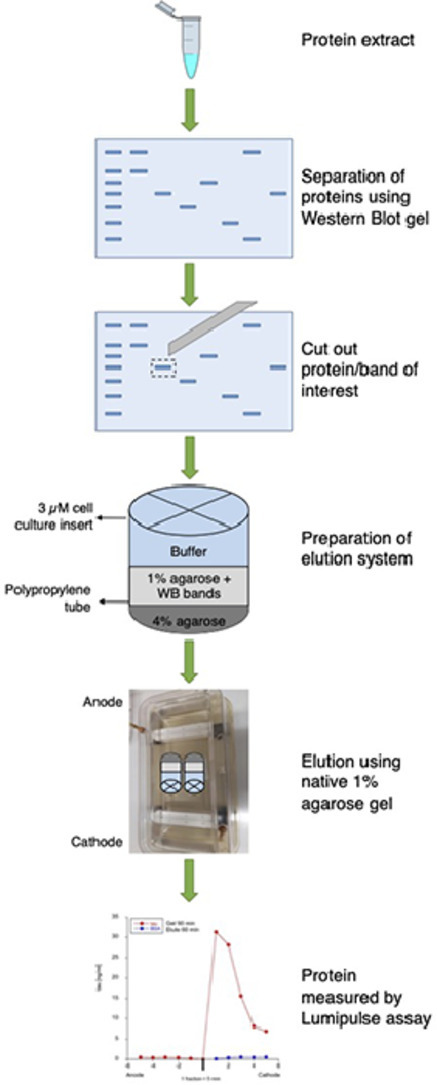

**Specifications table**

**Table:** 

Subject area;	Biochemistry, Genetics and Molecular Biology
More specific subject area;	Alzheimer´s disease, diagnosis, pathologies
Name of your method;	Western Agarose Native GELution (WANGEL)
Name and reference of original method;	Agarose native gel electrophoresis of proteins C. Li, T. Arakawa, Agarose native gel electrophoresis of proteins. Int. J. Biol. Macromol. 140 (2019, 668-671. https://doi.org/10.1016/j.ijbiomac.2019.08.066
Resource availability;	All resources used in this study are commercially available and the Lumipulse technology can be acquired from Fujirebio. More information can be found at https://www.fujirebio.com/en/products-solutions/lumipulse-g600ii.

## Method details

### Agarose gels

**Fig. 1:**
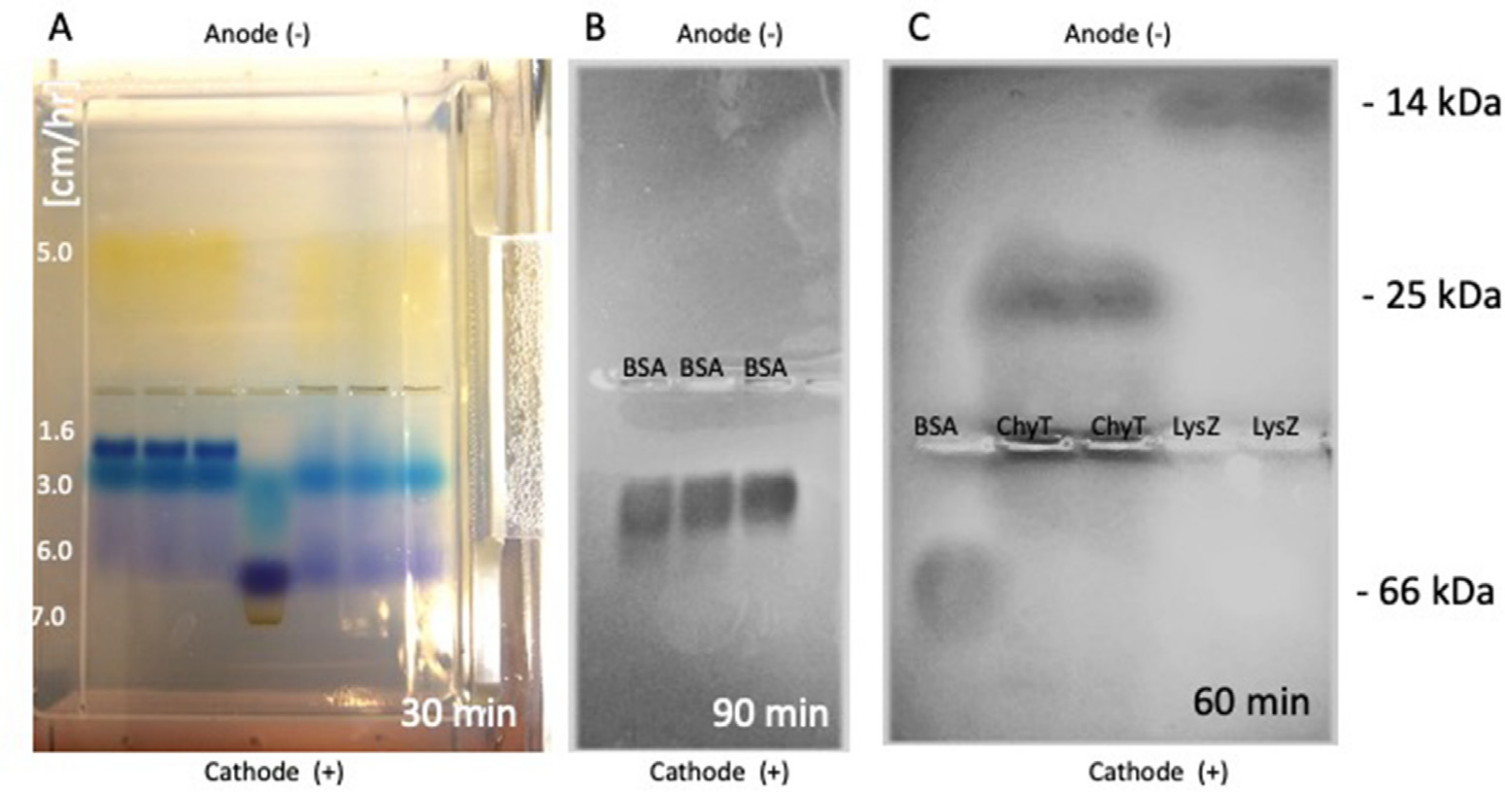
Set-up of horizontal agarose gel electrophoreses. A 1% agarose gel was prepared and the comb was placed in the middle of the gel. (A) Different color markers were loaded which migrated to the cathode within 30 minutes. The migration speed (cm/hr) is given on the left. (B) Bovine serum albumine (BSA, 20 µg/lane) migrates to the cathode within 90 min at a speed of 5 cm/hr. (C) As a control chymotrypsin (ChyT, 20 µg/lane) and lysozyme (LysZ, 20 µg/lane) migrate to the anode within 60 minutes. The molecular weight is given at the right side.

To make a 1% agarose gel, agarose (Sigma-Aldrich, A9539) was heated in the microwave (800W, 1 min), poured in a chamber, a comb (10 lanes) placed in the middle, and cooled (Fig. 1**A**). The gels were run in 100 mM MES (morpholino ethanesulfonic acid, Roth 4256.2) and 100 mM histidine (Roth, 1696.1) buffers (pH 6.1) for 100V, 250 mA, 40W and up to 90 min. Samples were loaded in bromophenolblue/ glycerine loading buffer (25 µl/gel). After separation, the gel was stained using PageBlue protein staining solution (ThermoFisher Scientific, 24620). To elute the specific samples from agarose gels, bands were cut from the gels and processed using the gel extraction kit (QuikPik, Stratagene, 400855) and electroeluted against cathode/anode for 60 min.

### Proteins/peptides

We tested bovine serum albumin (66 kDa, Serva, 11930.03), alpha-chymotrypsin type VII from bovine pancreas (25 kDa, Sigma-Aldrich, C3142) and lysozyme (14 kDa, Merck 528, 100000U/mg) for the characterization of agarose gels. For analysis of tau, 2N4R full-length tau (R&D, SP495) was used. For analysis of beta-amyloid human Aβ(42) and Aβ40) (both Innovagen, Sweden, SP-BA42 and SP-BA40 respectively) were used. Aβ42 was aggregated as detailed in a previous work from our lab [26].

### Human cerebrospinal fluid (CSF)

In our laboratory, we routinely analyze CSF from AD and Creutzfeld-Jacob disease (CJD) patients. For this present study, we used CSF of a CJD patient (#564, 2009). The CSF biomarker levels were 993 pg/ml of Aβ(42), 70 pg/ml of phospho-tau-181 and 3800 pg/ml of total tau.

### Mouse cortical extracts

Adult (6-month-old) control C57BL6 wild-type mice and transgenic AD mice were used. Transgenic APP_SweDI (expressing amyloid precursor protein (APP)) harboring the Swedish K670N/M671L, Dutch E693Q, and Iowa D694N mutations; C57BL/6-Tg (Thy1-APPSwDutIowa, BWevn/Mmjax) mice were used as reported previously by us [27]. These mice produce Aβ plaques at an age >6 months. Mice were sacrificed by decapitation, the cortex dissected and collected in tubes. The cortex was homogenized in 1 ml phosphate buffered saline (PBS) with a protease inhibitor cocktail (Sigma-Aldrich, P-8340) by sonication. This solution was centrifuged at 14,000xg for 20 min at 4°C and the supernatant was collected. Total protein amount was detected with Bradford assay with Coomassie brilliant blue G250 dye (Bio-Rad, #1610406).

### Western Blot of tau and Aβ(42)

**Fig. 2:**
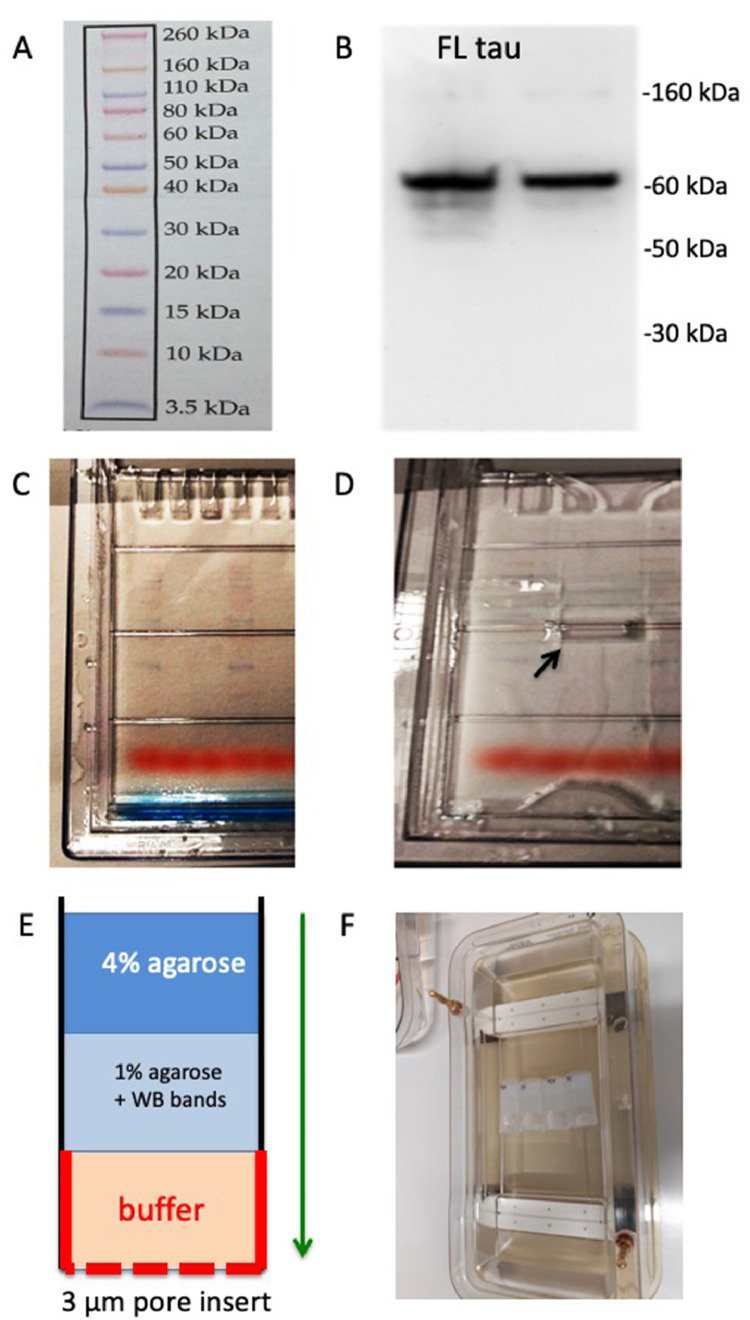
Combined Western Blot and agarose gel elution (WANGEL). (A) A colored molecular weight marker was loaded on a Western Blot gel and (B) full length tau (FLT tau) was detected on a Western Blot with a size of 60 kDa using the tau-5 antibody. (C) Tau protein was loaded on Western Blot gels incuding colored molecular weight markers and (D) the bands of interest (arrow) were cut out of the gel. (E) In order to elute tau on agarose gels, a tube was prepared with 4% agarose at the top, followed by 1% agarose with the cut bands embedded and loading buffer, then the collection buffer was added and the tube was closed with a 3 µm membrane insert. (F) These tubes were then added to the gel chamber and tau protein is eluted within 60 min at 100V.

Native Western Blots were performed as described previously [26]. Proteins (25 µl) were loaded on to the 10% Bis-Tris polyacrylamide gel (Invitrogen, NP0301BOX) with 5 μl of sample buffer. Electrophoresis was performed under native conditions for 30-40 min (tau) or 25 min (Aβ) at 200 V. Samples were electrotransferred onto PVDF membranes for 20 min at 25 V in semi-dry transfer cell (Thermo Scientific). Blotting was done with the WesternBreeze Chemiluminescent immunodetection system (Invitrogen). Blots were blocked for 30 min and incubated overnight on a shaker at 4°C with primary antibodies against tau (Tau-5, ThermoFisher Scientific, AHB0042, 1:1000) or human Aβ42) (BioLegend, SIG-39399, clone 6E10, 1:1000). Blots were incubated with alkaline phosphatase-conjugated secondary antibody (anti-mouse) for 30 min at room temperature. Following brief washing steps, blots were incubated in CDP-Star chemiluminescent substrate solution (Roche) for 15 min and visualized with a cooled CCD camera (SearchLight, ThermoFisher Scientific). A multi-colored molecular weight marker (Invitrogen, LC5800) was run as a control (Fig. 2**A**).

### Gel extraction and elution

After the Western Blots, different bands were dissected with a scalpel with a size between 0 and 80 kDa (Fig. 2 **C,D**). The proteins were eluted from the PAGE gels using a modified new method. For the elution, we used 2.5 ml sample vials (PE, 14 mm, Roth, 5863.1). One ml 4% agarose was pipetted at the bottom of the tubes and cooled. Then the Western Blot bands were carefully placed on top of the hardened agarose. Next, 1 ml of 1% handwarm agarose was pipetted onto the extracted bands and cooled to harden. Subsequently, the bottom of the vial was cut, and the tube closed with a Millicell culture insert (3 µm pore size, 12 mm, PITP01250, Merck). Following this, 1 ml of the MES/histidine buffer was carefully pipetted into the tube using a syringe and then 10 µl bromphenobuffer was pipetted into the tubes to follow up the elution (Fig. 2**E**). These tubes were placed into the agarose chamber and proteins eluted for 20-60 min to the cathode/anode (Fig. 2**F**). After the run, the buffer with the eluted proteins was collected and analyzed by the Lumipulse immunoassay.

### Lumipulse tau detection

Total tau and Aβ42) and (40) levels were measured using automated Lumipulse technology (Fujirebio G600II); In brief, the Lumipulse G600II is started and prepared with all sufficient reagents (wash solution, substrate, tips, dilution, a.d.) and the single cartridges for each marker are loaded to the system. The G600II is an automated robotor system and designed to perform all steps itself. Samples (500 µl) are pipetted into Hitachi cups, and added to the racks, the system is started and values can be read after 35 min. Standard curves and quality controls verify the correct values. The system works on basis of an immunologcal reaction and is a 2-step chemicluminescence assay.

## Method validation

### Characterization of migration in the 1% agarose gels

Different color markers (bromphenolblue, mid green) were loaded which migrated to the cathode within 30 min. (Fig. 1**A**). Bovine serum albumine (20 µg/lane) migrated to the cathode within 90 min at a speed of 5 cm/h (Fig. 1**B**). Chymotrypsin (20 µg/lane) and lysozyme (LysZ, 20 µg/lane) migrated to the anode within 60 min (Fig. 1**C**).

### Characterization of tau in 1% agarose gels

**Fig. 3:**
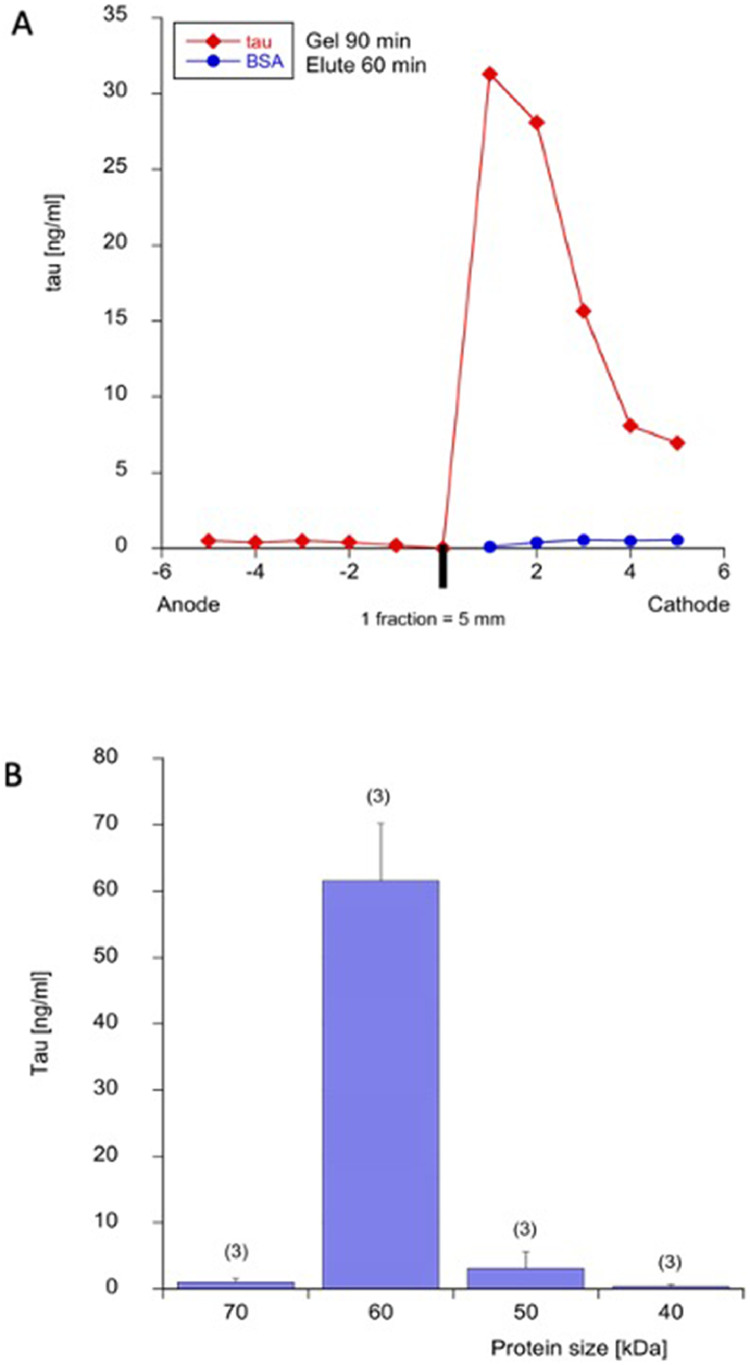
Elution of tau from Western Blots linked to agarose gels (WANGEL). (A) Tau protein (100 ng/lane) was loaded to 1% agarose gels and then the gel was run at 100V for 90 min. Agarose fragments were cut and eluted within 60 min. Tau protein (red squares) migrates to the cathode and is seen within the first 2 (five mm) fractions. BSA (blue circles) is loaded as a negative control. (B) Tau protein (100 ng/lane) was separated on a Western Blot gel, then bands with a size of 70-60-50-40 kDa are cut, and tau eluted from agarose gels. Note that tau elutes as a 60 kDa protein from Western-agarose gels. Values are given as tau levels in ng/ml, then detected by Lumipulse technology (n=3 independent experiments).

Full-length tau is a 60 kDa protein that was detected as a single band on a Western Blot (Fig. 2**B**). Tau was verified on the PAGE gels by colored molecular markers (Fig. 2**C**) and the bands cut out with a razor (Fig. 2**D**). In order to elute tau on agarose gels, the cut bands with 60 kDa were eluted from special agarose-embedded tubes (Fig. 2**E**) and eluted in the agarose gel chamber (Fig. 2**F**). With the 1% agarose gels, tau migrated to the cathode and not the anode within 90 min in the first two fractions (Fig. 3**A**). After Western Blot gel separation and elution in agarose (WANGEL) via the cathode, tau was measured by Lumipulse immunoassay as a 60 kDa protein (Fig. 3**B**). BSA served as a negative control for the assay (Fig. 3**A**).

### Characterization of mouse cortex and human CSF

**Fig. 4:**
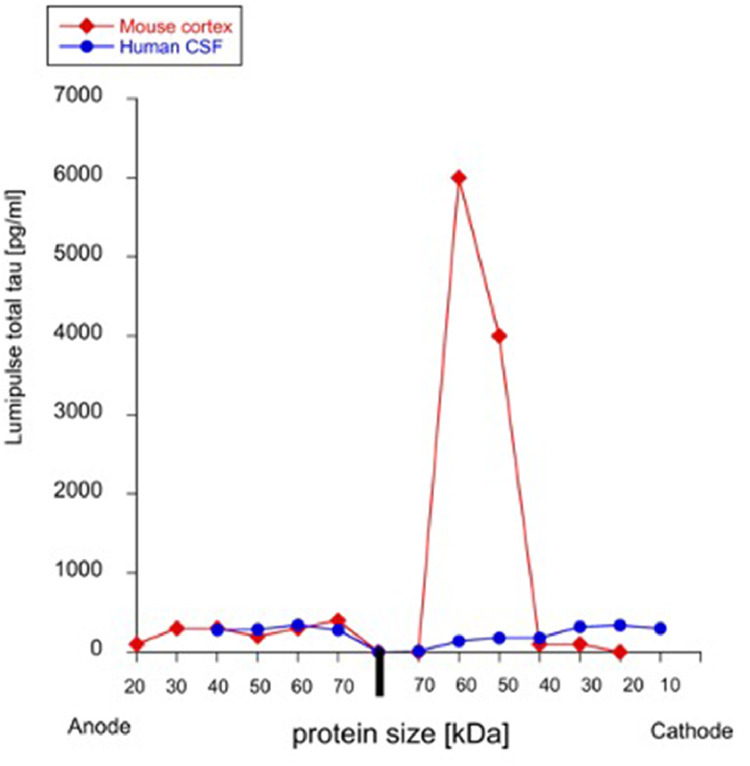
Characterization of tau from mouse cortex and human cerebrospinal fluid (CSF). Mouse cortex extracts from wildtype mice (red squares) and human CSF (blue circles) were separated on a Western Blot gel (40 min), then bands with a size between 70 and 10 kDa were cut, and tau eluted from agarose gels (100V, 60 min) and tau was detected using Lumipulse technology. Note that mouse cortical tau elutes as a 60 kDa protein from Western-Agarose gels. Cortex was extracted from wildtype mice and give a protein concentration of 2.1 mg/ml and 616 ng tau/mg (Lumipulse); note that 4 lanes (each 20 µl) were pooled. Human CSF was tested from a well characterized Creutzfeld-Jacob disease (CJD) patient with 3,800 pg/ml total tau; note that 8 lanes (each 20 µl) were pooled but the levels were below detection limit.

Mouse corteical extracts from wild-type mice were separated on a Western Blot gel, and different molecular weight bands extracted and eluted. Tau was seen as a large 60 kDa protein eluted from 1% agarose (WANGEL) via the cathode and measured in the Lumipulse assay (Fig. 4). The analysis of a CSF from a CJD patient (a pool of 8 lanes) could not detect tau as the detection levels were too low (Fig. 4).

### Characterization of beta-amyloid(42) from WANGEL

**Fig. 5:**
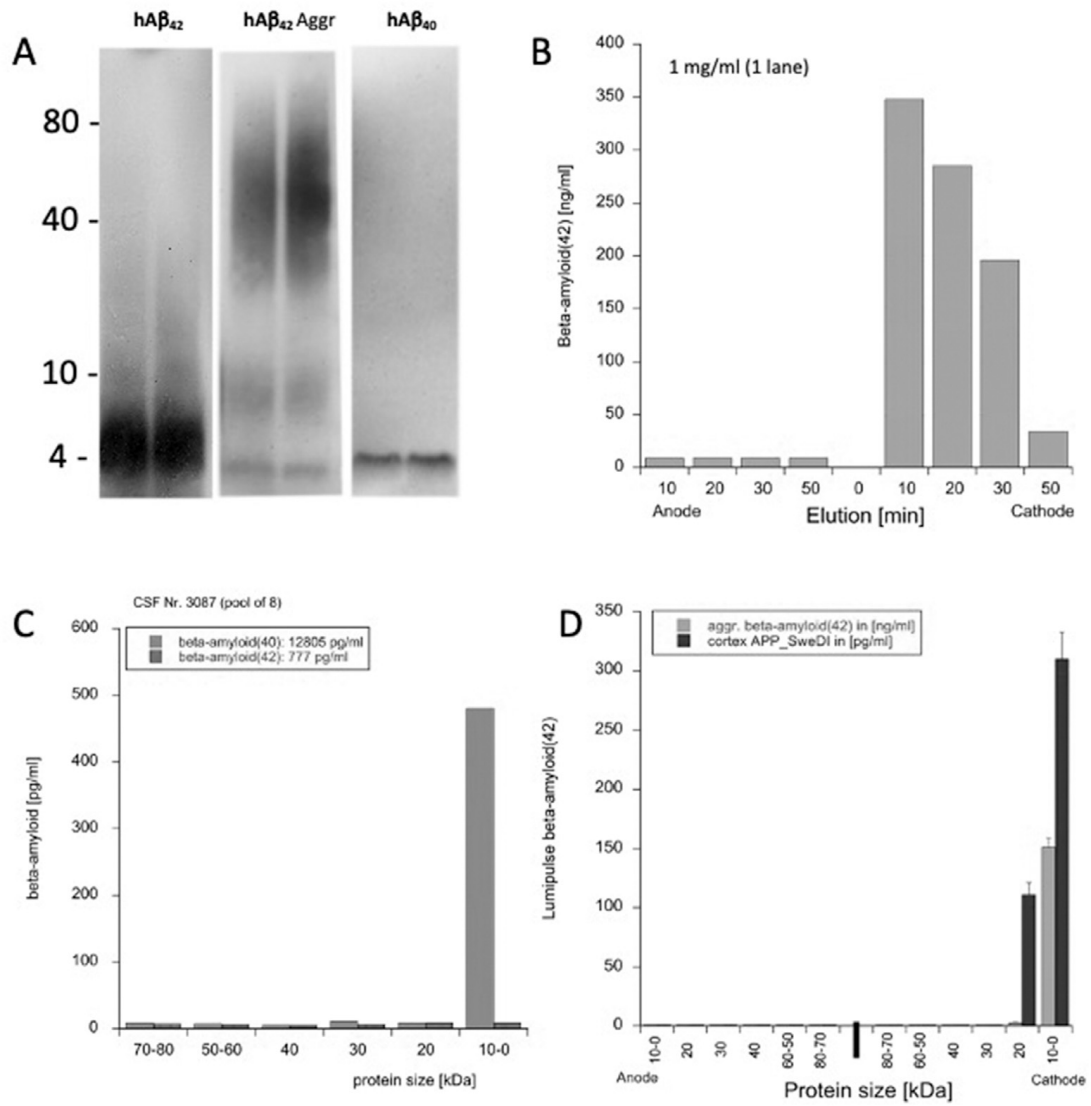
Characerization of beta-amyloid (Aβ) from WANGEL. (A) Aβ(42) and Aβ(40) standards (500 ng/lane) were loaded on PAGE gels and characterized by Western Blots. Note that Aβ is a 4 kDa peptide. Further aggregated Aβ(42) appears as a high molecular smear on the gel. (B) Aβ(42) was separated on native PAGE gels, 0-10 kDa bands cut and eluted in the agarose gel for 10-40 min. Note that Aβ elutes after 10-20 min when run against the cathode. (C) Cerebrospinal fluid was separated and eluted and Aβ(40)  was detectable as a 4 kDa peptide; Aβ(42) was below the detection level. (D) Elution of aggregated Aβ(42) from WANGEL shows that the Lumipulse assay can only detect the 4 kDa species but not higher molecular weight species. Interestingly in a cortical extract taken from transgenic APP_SweDI mice Aβ was detectable as a 4kDa species but also higher low molecular oligomeric aggregates (up to 20 kDa) were found.

Aβ(42) and Aβ(40) were found as a 4 kDa peptide on Western Blots and aggregated Aβ42) appeared as a high molecular smear on the gel (Fig. 5**A**). Aβ42) was separated on native PAGE gels and eluted via the cathode in 1% agarose within 10-20 min **(**Fig. 5**B).** In order to characterize Aβ in CSF, CSF was eluted from Western Blots and Aβ40) but not Aβ42) was detected by Lumipulse. Aggregated human Aβ (42) appears as a high molecular weight species smear on the Western Blot (Fig. 5**A)**, however, in the eluted fractions (after WANGEL) only a 4 kDa species was detectable by Lumipulse assay which migrated to the cathode but not anode (Fig. 5**D**). Cerebrospinal fluid was separated and eluted and Ab(40)  was detectable as a 4 kDa peptide; Ab(42) was below the detection level (Fug. 5C). In a cortical extract taken from transgenic mice (APP_SweDI) the Aβ(42) was detectable as a native peptide but also as small oligmeric aggregates (Fig. 5**D**).

Taken together, we present a novel extraction-elution model (WANGEL), where we first separate tau and beta-amyloid on a commercial Western PAGE gel and then elute into a native agarose gel. Using this method, we could successfully detect a 60 kDa tau protein and a 4 kDa beta-amyloid peptide through the commercial Lumipulse (Fujirebio) assay system. This method is fast, cheap and can be easily adapted in any laboratory. Many cut out lanes can be pooled and eluted and thus, enable to concentrate the sample. We demonstrate that Lumipulse can detect mouse tau, can analyze mouse cortical extracts and it can be useful to characterize distinct molecular weight species.

### Alzheimer's disease pathology

The life expectancy of humans has markedly increased over the last 100 years. As age is the main risk factor for Alzheimer's disease (AD), the number of patients suffering from AD and mixed forms of dementia will dramatically increase over the next 50 years. Latest reports forecast that there will be about 152 million AD-dementia patients worldwide in 2050, which is an overwhelming increase from 57 million AD-dementia patients in 2019 [1]. The majority of AD cases are sporadic in nature and the etiology of the disease arises from both genetic and non-genetic factors (at least 97% of cases). Sporadic AD is characterized by two major pathologies: the β-amyloid (Aβ) pathology leading to extracellular plaques and the tau pathology, including hyperphosphorylated tau, resulting in intraneuronal neurofibrillary tangles [2], [3], [4], [5], [6], [7]. Accompanying pathologies include cell death of cholinergic neurons, reactive astrogliosis, microglial activation, inflammation and cerebrovascular damage, specifically blood-brain barrier damage [8], [9], [10].

### Tau and beta-amyloid in CSF

The analysis of cerebrospinal fluid (CSF) is certainly a state-of-the-art technique for AD diagnostics using human fluids [11], [12], [13]. The analysis of Aβ (with 40 and 42 amino acids), total tau and phosphorylated tau (at position 181) is internationally recognized and established, including in our research group [14]. The CSF control levels of these biomarkers are as follows: ∼800 pg/ml of Aβ(42), ∼10,000 pg/ml of Aβ(40), ∼300 pg/ml of total tau and ∼30 pg/ml of phospho-tau-181. The combined analysis of all four biomarkers in CSF permits the diagnosis of AD with >90% specificity and sensitivity [15], [16], [17], [18].

### Lumipulse detection of tau and beta-amyloid

Immunoassays are an advanced and effective technique to detect low levels of tau and Aβ. Although many immunoassays being globally available, the use of the Innogenetics/Fujirebio assay is universally documented and accepted [19], [20]. While enzyme-linked immunoassay (ELISA) systems are a routinely utilized method, they tend to be time consuming (1-2 days). Recent advances in automated systems enable the detection of tau and Aβ within 35 minutes. This Lumipulse G600II technology is based on an antibody detection system and utilizes a simple and easy cartridge system. Lumipulse technology (https://www.fujirebio.com/en/products-solutions/lumipulse-g600ii) is well established in our lab to routinely analyze CSF biomarkers for AD diagnosis. However, this system detects only total fluid biomarker levels and does not differentiate between different molecular weight species. Moreover, this system has been validated for CSF detection, but not for other fluids, such as plasma, saliva or mouse cortical extracts.

### Western Blot of tau and beta-amyloid

Western Blots are a gold standard approach when it comes to detecting different molecular weight species [21]. Proteins are usually separated on native or denatured polyacrylamide gels (PAGE) and detected using an antibody-enzyme system (e.g. enhanced chemiluminescence). Using Western Blots, it is acknowledged that full length tau is a 60 kDa protein and Aβ(42) and Aβ(40) are small 4 kDa peptides. The Aβ(42) peptides can aggregate and show a smear of higher molecular weight species. In addition, tau is present as smaller tau fragments in CSF [22]. Furthermore, post-analysis of Western Blot eluates through ELISA or mass spectrometry is limited owing to the toxic properties of polyacrylamide.

### Agarose gels and elution problem

Agarose gels are a simple and cheap way to separate different molecules, not only for separation of DNA but also RNA molecules (e.g. formaldehyde gels) [23]. It is reported that 1% agarose gels are suitable to separate and analyze different high molecular weight proteins, such as bovine serum albumin, lysozyme or chymotrypsin [24]. Distinct proteins can be separated by transfer to the cathode or anode. DNA/RNA from such agarose gels is eluted to isolate cloning/PCR fragments for further cloning or characterization [25]. However, the elution of proteins/peptides from native agarose gels is not well characterized.

## Ethics statements

All animal experiments with mice were approved by the Austrian Ministry of Science and Research (66.011/0055-WF/V/3b/2017) and conformed to the Austrian guidelines on animal welfare and experimentation.

## Funding

This study was supported by the Austrian Science Funds (P32558-B).

## CRediT authorship contribution statement

**Dhwani S. Korde:** Writing – review & editing, Visualization. **Christian Humpel:** Conceptualization, Methodology, Writing – original draft, Funding acquisition.

## Declaration of Competing Interest

The authors declare that they have no known competing financial interests or personal relationships that could have appeared to influence the work reported in this paper.
